# Targeting ion channel networks in diabetic kidney disease: from molecular crosstalk to precision therapeutics and clinical innovation

**DOI:** 10.3389/fmed.2025.1607701

**Published:** 2025-06-26

**Authors:** Wenfeng Wang, Bi Ke, Chen Wang, Xiaojing Xiong, Xiuyuan Feng, Hua Yan

**Affiliations:** ^1^Department of Cardiology, Wuhan Asia General Hospital Affiliated to Wuhan University of Science and Technology, Wuhan, Hubei, China; ^2^Department of Cardiology, Ezhou Central Hospital, Ezhou, Hubei, China

**Keywords:** ion channels, diabetic kidney disease, targeted therapy, precision medicine, sodium-glucose cotransporter 2

## Abstract

Diabetic kidney disease (DKD), a major microvascular complication of diabetes, is closely associated with functional imbalances in ion channels regulating sodium (Na^+^), calcium (Ca^2+^), potassium (K^+^), and chloride (Cl^–^). This review systematically examines the roles of ion channels in glomerular filtration barrier dysfunction, tubular reabsorption, and fibrotic processes in DKD, with emphasis on the pathological relevance of sodium-glucose cotransporter 2 (SGLT2), epithelial sodium channels (ENaC), transient receptor potential (TRP) channels, chloride channels, aquaporins (AQPs), and PIEZO channels. We further evaluate the clinical efficacy and challenges of ion channel-targeted therapies, including SGLT2 inhibitors and mineralocorticoid receptor antagonists. Emerging strategies integrating ion channel omics, machine learning, engineered biomaterials, and exosome-based delivery systems are proposed to shift DKD treatment paradigms from disease progression delay to pathological reversal. Interdisciplinary collaboration is critical to achieving personalized precision medicine, offering novel perspectives for DKD diagnosis and management.

## 1 Introduction

Diabetic kidney disease (DKD), one of the most severe microvascular complications of diabetes, has become the leading cause of end-stage renal disease (ESRD) worldwide. Epidemiological data indicate that approximately 40% of diabetic patients develop chronic kidney disease (CKD), with type 2 diabetes posing a higher risk. Between 1990 and 2017, diabetes-related CKD cases surged by 74%, from 1.4 million to 2.4 million (1–3). The proportion of ESRD cases attributed to diabetes increased from 19% in 2000 to 29.7% in 2015, with further increases anticipated (3). The Western Pacific region (e.g., China, Japan) and the United States exhibit the highest incidence of diabetes-related ESRD, with rates 2–3 times greater than those in Europe (4). China alone has over 110 million diabetic patients, of whom 32.5% with type 2 diabetes develop CKD, translating to over 30 million DKD cases. However, awareness and screening rates remain alarmingly low at 26% and < 55%, respectively (4–7). DKD now represents the primary etiology of CKD-related hospitalizations in China and dominates new hemodialysis cases. These trends underscore the immense global healthcare and economic burden of CKD.

The core pathological features of DKD include glomerular hyperfiltration, podocyte injury, tubulointerstitial fibrosis, and chronic inflammatory microenvironments. Disease progression is driven not only by metabolic dysregulation but also by complex molecular network imbalances (8–12). While conventional therapies targeting glycemic control, blood pressure management, and renin-angiotensin-aldosterone system (RAAS) inhibition remain foundational, approximately 30% of DKD patients experience relentless renal function decline despite intensive treatment, highlighting the urgent need for novel therapeutic targets (7–9, 13).

This review specifically addresses this therapeutic gap by examining the pivotal role of ion channel dysfunction in DKD pathogenesis—a mechanism insufficiently targeted by current treatments. Recent studies implicate ion channel dysfunction as a pivotal driver of DKD pathogenesis. The kidney, a hub for ion transport, relies on precise regulation of Na^+^, K^+^, and Ca^2+^ channels. For instance, SGLT2 overexpression in proximal tubules exacerbates Na^+^ and glucose reabsorption, inducing glomerular hypertension and oxidative stress, thereby accelerating podocyte detachment and basement membrane thickening (14). Conversely, aberrant ENaC activation in distal nephrons promotes Na^+^ retention, hypertension, and tubulointerstitial fibrosis via a “metabolic-hemodynamic” vicious cycle (15–17). Additionally, pathological Ca^2+^ influx mediated by TRP channels (e.g., TRPC6, TRPV4) triggers podocyte cytoskeletal remodeling, mitochondrial dysfunction, and apoptotic signaling, directly contributing to proteinuria (17–19).

Mounting evidence demonstrates that SGLT2 inhibitors confer renal protection through three distinct yet complementary mechanisms: First, by suppressing excessive glucose and sodium reabsorption in proximal tubules, these agents restore tubuloglomerular feedback and ameliorate glomerular hyperfiltration (20). Second, they modulate tissue oxygenation homeostasis while attenuating inflammatory and fibrotic pathways (21). Third, they promote blood pressure reduction (mean decrease 3.3 mmHg) and vascular resistance improvement through enhanced urinary sodium and glucose excretion (20). Clinical trials confirm that SGLT2 inhibitors reduce the risk of composite renal endpoints by 30% (including end-stage renal disease and serum creatinine doubling), accompanied by modest reductions in HbA1c (0.25%) and body weight (0.8 kg) (20). Notably, these nephroprotective effects appear mediated through common mechanisms in both diabetic and non-diabetic CKD populations, while providing additional cardiovascular benefits including amelioration of atherosclerosis and mitigation of uremic toxin-induced cardiac damage (22, 23).

Representative SGLT2 inhibitors (e.g., dapagliflozin) exert their effects by reducing intraglomerular pressure and restoring tubuloglomerular feedback, thereby optimizing renal energy metabolism. The DAPA-CKD trial demonstrated that dapagliflozin could delay ESRD progression by 6.6 years (24–26). Similarly, the selective mineralocorticoid receptor antagonist finerenone, by inhibiting ENaC overactivation, achieves a 32% reduction in urinary albumin-to-creatinine ratio (UACR) and a 23% decrease in kidney-related events, marking a therapeutic breakthrough in DKD management (27). Emerging therapeutic strategies, including TRPC5 inhibitors, have shown promise in reversing podocyte injury in preclinical models (28, 29), highlighting the substantial clinical potential of ion channel-targeted therapies. However, challenges persist due to the complexity and tissue specificity of ion channel regulation. For example, SGLT2 inhibitors may induce euglycemic ketoacidosis, while ENaC antagonists require careful monitoring of hyperkalemia risk (30). Future research must integrate single-cell omics and structural biology (e.g., cryo-electron microscopy) to elucidate dynamic ion channel expression profiles across renal cell populations and develop subtype-specific modulators. Recent structural resolution of the Cav2.1 calcium channel subunit (31) provides a molecular blueprint for selective drug design, a strategy applicable to DKD-related channels.

Ion channel dysregulation exhibits intricate associations with inflammatory and fibrotic pathways in DKD. A comprehensive elucidation of these molecular networks will enable targeted therapeutic interventions to achieve disease remission. Our purpose is thus threefold: (1) Elucidate molecular linkages between ion channel dysregulation and DKD progression; (2) Evaluate clinical challenges in translating channel-targeted therapies; (3) Propose precision strategies (e.g., single-cell omics, structural pharmacology) to develop subtype-specific modulators that mitigate off-target risks while maximizing renoprotection. By integrating these insights, we aim to advance ion channel therapeutics from disease management toward personalized, pathology-reversing regimens for DKD.

## 2 Literature search strategies

We conducted a systematic narrative literature review utilizing PubMed, Web of Science, Google Scholar, and EMBASE databases (2022–2025) with the following search terms: “ion channels,” “diabetic kidney disease,” “SGLT2 inhibitors,” “TRP channels,” and “precision therapy.” The review incorporated preclinical studies, clinical trials, and relevant review articles. Furthermore, we critically examine future research directions and clinical prospects regarding ion channel modulation in DKD, while providing expert perspectives. This review synthesizes current evidence concerning ion channel mechanisms, therapeutic targets, and innovative multidisciplinary approaches (including multi-omics integration, machine learning applications, engineered nanocarrier systems, and structural pharmacology) to transform DKD treatment paradigms from disease progression delay to potential pathological reversal.

## 3 Pathogenesis and current research in DKD

**FIGURE 1:**
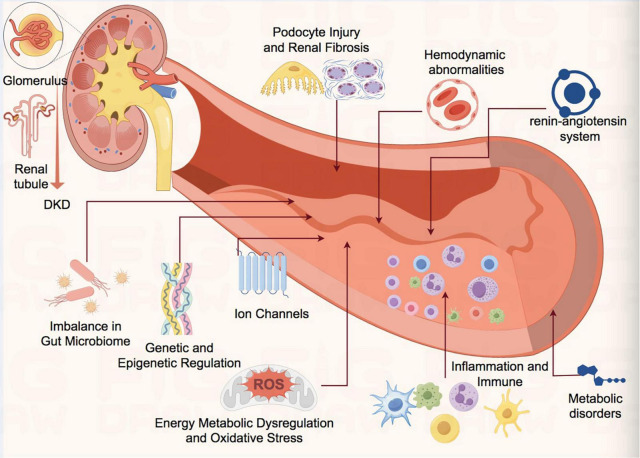
Schematic diagram of pathophysiological mechanism of DKD. DKD, diabetic kidney disease; ROS, reactive oxygen species.

The pathogenesis of DKD involves multifactorial interactions among energy metabolic dysregulation, oxidative stress, inflammatory responses, fibrosis, genetic susceptibility, renin angiotensin system (RAS) intestinal flora and metabolites forming a complex “metabolic-inflammatory-fibrotic” cascade network. Current mechanistic research has shifted from a singular metabolic perspective to multidimensional network regulation, integrating interdisciplinary technologies to dissect the “gene-environment-Gut Microbiota-metabolism” interplay, which is critical for elucidating the underlying mechanisms of DKD (Figure 1).

### 3.1 Energy metabolic dysregulation and oxidative stress

Hyperglycemia activates the polyol pathway, protein kinase C (PKC) signaling, and accumulation of advanced glycation end products (AGEs), inducing renal oxidative stress and energy metabolism abnormalities (32–37). AGE binding to its receptor (RAGE) activates NADPH oxidase, leading to reactive oxygen species (ROS) overproduction, mitochondrial dysfunction, and DNA damage, thereby promoting podocyte apoptosis and glomerular basement membrane thickening (35–38). Additionally, hyperglycemia suppresses ATP-sensitive potassium (KATP) channels, exacerbating membrane depolarization and calcium overload, further damaging tubular epithelial cells (39).

### 3.2 Inflammation and immune dysregulation

Chronic inflammation is a key driver of DKD progression. Hyperglycemia activates NF-κB and TGF-β1 pathways, promoting macrophage infiltration and release of pro-inflammatory cytokines (e.g., TNF-α, IL-6, IL-1β), thereby fostering an inflammatory microenvironment (39–41). Overexpression of monocyte chemoattractant protein-1 (MCP-1) and fibronectin (FN) accelerates glomerulosclerosis, while Th17/Treg cell imbalance impairs anti-inflammatory responses, perpetuating a vicious cycle (42–44).

### 3.3 Podocyte injury and renal fibrosis

Podocytes, critical components of the glomerular filtration barrier, undergo injury that underlies proteinuria and renal functional loss (45, 46). Hyperglycemia induces aberrant expression of long non-coding RNAs (e.g., lncRNA evf-2) in podocytes, which bind heterogeneous nuclear ribonucleoprotein U (hnRNPU) to regulate cell cycle re-entry and inflammatory responses, exacerbating podocyte detachment (47). Pathological calcium influx mediated by transient receptor potential (TRP) channels (e.g., TRPC6, TRPV4) triggers podocyte cytoskeletal remodeling and apoptosis, further disrupting the filtration barrier (48–50). TGF-β1 signaling activation drives epithelial-mesenchymal transition (EMT), promoting tubulointerstitial fibrosis and extracellular matrix (ECM) deposition (51).

### 3.4 Genetic and epigenetic regulation

Genetic polymorphisms (e.g., ACE insertion/deletion polymorphism) and epigenetic modifications (e.g., DNA methylation, histone acetylation) significantly influence DKD susceptibility (52–58). Genetic predispositions conferred by polymorphisms in ELMO1, AGTR1, and P2 × 7 also play critical roles in DKD pathogenesis (53–55). Furthermore, lncRNAs (e.g., H19, MALAT1) modulate chromatin remodeling to influence inflammation-related gene expression, while miRNAs (e.g., miR-21, miR-29) target fibrotic pathway genes, contributing to renal fibrosis (56–62). These findings underscore the pivotal role of genetic factors in DKD development.

### 3.5 Multiple regulatory effects of renin angiotensin system (RAS)

The renin-angiotensin(Ang)-aldosterone system (RAAS) plays a critical role in the pathogenesis of both acute and chronic kidney diseases.

Recent studies have demonstrated that abnormal activation of the renin-angiotensin system (RAS) not only promotes glomerular injury through the classical Ang II/AT1R axis but also involves novel mechanisms including RhoA/ROCK signaling pathway-mediated cytoskeletal remodeling (63–65). RAS activation upregulates the RhoA/ROCK signaling pathway, resulting in increased endothelial permeability and cytoskeletal reorganization. Similar mechanisms may be triggered by mechanical stress (such as glomerular hypertension) in DKD. Inhibition of RhoA/ROCK can reduce podocyte detachment and mesangial matrix deposition, indicating this pathway serves as a crucial downstream effector of RAS (63). Additionally, emerging research suggests that the histone deacetylase SIRT6 can attenuate RAS-induced profibrotic and oxidative stress responses by inhibiting the Wnt/β-catenin signaling pathway, thereby ameliorating podocyte injury and glomerulosclerosis (66). Ellagic acid has been shown to significantly reduce TNF-α levels in cisplatin-induced nephrotoxicity models while suppressing the ERK1/2-NF-κB inflammatory pathway through SIRT6 activation (67). Additionally, non-classical RAS components contribute to renal injury. The imbalance of the Ang-(1–7)/Mas axis exacerbates oxidative stress, while aldosterone promotes fibrosis through upregulation of ENaC and TGF-β1. Novel non-steroidal mineralocorticoid receptor antagonists (e.g., Finerenone) can partially reverse these pathological processes (68).

### 3.6 Mechanisms of interaction between gut microbiota dysbiosis and the gut-kidney axis

The gut microbiota, as the largest microbial ecosystem in the human body, plays a pivotal role in host metabolic processes. Dysbiosis of the gut microbiota is closely associated with metabolic disorders such as diabetes. Patients with diabetic nephropathy (DN) exhibit significant differences in both the compositional distribution of gut microbiota and the levels of gut-derived metabolites compared to healthy individuals (69). Microbial metabolites, including short-chain fatty acids (SCFAs), bile acids, hydrogen sulfide, and uremic toxins, function as chemical messengers (70). These metabolites influence renal function in DN by modulating immune responses, inflammatory cascades, and oxidative stress pathways.

The latest research has revealed the key role of intestinal flora imbalance in DKD and potential intervention strategies: SGLT2 inhibitors (such as dapagliflozin) can delay the progress of DKD by regulating bile acid metabolism spectrum, enhancing antioxidant capacity, and dynamically improving the structure of intestinal flora (71). Additionally, probiotic intervention (such as lactic acid bacteria) can produce indole derivatives through tryptophan metabolism, inhibit the activation of aryl hydrocarbon receptor (AHR) pathway, reduce glomerular basement membrane damage and podocyte apoptosis, and provide a new treatment approach for membranous nephropathy complicated with DKD (72). Clinical studies further found that the characteristics of intestinal flora in patients with DKD (especially in ESRD stage) were significantly correlated with deterioration of renal function (such as decreased eGFR, accumulation of uremic toxins) and psychological distress (73). These findings provide a theoretical foundation for multi-dimensional interventions targeting the gut-kidney axis, including microbiota remodeling and metabolite regulation.

### 3.7 Interorgan regulatory networks in metabolic disorders

Metabolic disorders such as diabetes are characterized by nutrient metabolism dysregulation. Recent studies highlight the critical role of amino acids in metabolic homeostasis. Branched-chain amino acids (BCAAs)—leucine, isoleucine, and valine—are essential amino acids with potent metabolic regulatory effects. Elevated plasma BCAA levels in DKD patients, coupled with downregulation of catabolic genes (e.g., BCKDK), exacerbate mitochondrial dysfunction and oxidative stress via AMPK pathway suppression (74, 75).

Furthermore, the latest research reveals that intestinal flora dysbiosis in DKD patients leads to abnormal metabolite profiles, manifested by reduced short-chain fatty acids (such as acetic acid and propionic acid) and accumulated uremic toxins (such as TMAO and indoxyl sulfate). These changes aggravate renal inflammation and fibrosis by activating the AHR pathway and pro-inflammatory factors (IL-6 and TNF-α) (72, 75). SGLT2 inhibitors (such as dapagliflozin) can enhance antioxidant capacity and delay DKD progression by regulating bile acid metabolic profiles and maintaining bacterial population balance. Simultaneously, probiotics (such as lactic acid bacteria) inhibit the AHR pathway through tryptophan-derived indole metabolites, thereby reducing kidney injury (66, 71, 72). Traditional Chinese medicine (such as Tangshen formula) promotes SCFA production and reduces uremic toxins by remodeling microbial composition (including Firmicutes/Bacteroidetes ratio regulation), with mechanisms involving multi-target modulation of the gut-kidney axis (72). Multigroup analyses further confirm that Blautia abundance and bile acid dysmetabolism in hypertensive DKD models drive disease progression via the microbial-intestinal-metabolic axis, suggesting that targeted microbial metabolic microenvironment interventions (such as FMT and metabolite supplementation) represent novel therapeutic directions for DKD (73). Future studies should employ multi-omics technologies to analyze flora-metabolite interaction networks for optimizing precision intervention strategies.

## 4 Role of ion channels in DKD pathogenesis and targeted intervention strategies: from mechanistic insights to precision therapy

Ion channels, as core regulators of renal electrolyte balance and signal transduction, are critically implicated in the pathological progression of DKD (76–81). Dysfunction of ion channels in DKD exhibits spatiotemporal specificity and network-dependent regulation, intricately intertwined with metabolic disturbances, oxidative stress, inflammatory fibrosis, and podocyte injury. Hyperglycemia-induced activation of the polyol pathway, protein kinase C (PKC), and advanced glycation end product (AGE) signaling initiates a vicious cycle of ion channel dysfunction and oxidative stress. This section systematically examines the physiological roles and dysregulation of sodium (Na^+^), calcium (Ca^2+^), potassium (K^+^), and chloride (Cl^–^) channels across nephron segments in DKD, integrating recent preclinical and clinical advances. The following table shows the latest studies on i*on channel-targeted therapies in DKD* models and their corresponding mechanisms in DKD (Table 1).

**TABLE 1:** Ion channel-targeted therapies in DKD models.

Drug/target	Model (cell/animal)	Dose/concentration	Key mechanism	References
Dapagliflozin (SGLT2i)	db/db mice, STZ-induced mice, human podocytes	1 mg/kg, 10 μM	Modulates ERRα-ACOX1 axis-mediated lipid metabolism	(163)
	STZ-induced mice	10 mg⋅kg	Inhibits ferroptosis and ameliorates renal fibrosis	(164)
	REDD1-knockout (PodKO) STZ-induced mice	1 mg/kg, 5, 10, or 20 μM	Reduces renal REDD1 protein abundance and immune cell infiltration	(165)
	HFD/STZ-induced mice, HK-2 cells	1 mg/kg	Alleviates renal inflammation/fibrosis via gut microbiota remodeling	(166)
	*db/db mice; STZ -*induced Sprague-Dawley (SD) rats, BV2 microglia	1 mg/kg, 0.28 mg/3 μL	Upregulates MCPIP1 to improve hypothalamic neuroinflammation	(167)
Empagliflozin (SGLT2i)	STZ-induced CD-1 mice and HFD-fed C57BL/6 mice, HK-2 cells	10 mg/kg, 1 mM	Downregulates renal tubular PKM2 to reduce fibrosis	(168)
	db/db mice	0.045%	Blocks AGE-RAGE axis-mediated metabolic derangements	(169)
	STZ	35 mg/kg	Regulates redox profile and inhibits pyroptosis	(170)
	db/db	15 mg/kg	Improves mitochondrial quality control via Prdx3-PINK1 pathway	(171)
	SGLT2 knockout STZ/HFD-induced diabetic	10 mg/kg	Rebalancing mitochondria-associated endoplasmic reticulum membranes	(172)
Finerenone (MRA)	*db*/*db* mice,HFD/STZ-Induced *C5aR1* KO mice,RAW 264.7, mouse macrophage cell line	10 mg/kg, 5 mM	Suppresses C5a-C5aR1 axis activation in macrophages	(103)
	HFD/STZ mice; Human kidney proximal tubular epithelial cells (HK-2 cells)	3 mg/kg, 5 mM	Enhances mitochondrial function via PI3K/Akt/eNOS signaling	(173)
TRPC6 inhibition	Trpc6-knockout mice, STZ mice	N/A	Trpc6 may play an important role in contributing to the interaction of diabetes and hypertension to promote kidney injury.	(162)
CLCA1/TMEM16A siRNA	db/db mice, MCT cells	N/A	Links H_2_S deficiency to CLCA1/TMEM16A-mediated Cl^–^ current dysregulation	(116)
Piezo1 inhibition	Piezo1-knockout STZ/HFD mice	N/A	Promotes podocyte injury via Piezo1/NFATc1/TRPC6 axis	(133)

### 4.1 Sodium channels

Sodium channels are pivotal molecular components for maintaining systemic sodium homeostasis and blood pressure regulation, distributed across distinct nephron segments to execute specialized functions. This section focuses on sodium channels critically involved in DKD pathophysiology.

#### 4.1.1 Sodium-glucose cotransporter 2 (SGLT2)

Sodium-glucose cotransporters (SGLTs) in renal proximal tubules, particularly SGLT2, play a central role in glucose homeostasis by reabsorbing 90% of filtered glucose. SGLT2, localized to the apical membrane of proximal tubule S1/S2 segments, actively transports one glucose molecule with two sodium ions, serving as the rate-limiting step for glomerular glucose reabsorption. In DKD, hyperglycemia upregulates SGLT2 expression via hypoxia-inducible factor-1α (HIF-1α)- and reactive oxygen species (ROS)-dependent transcriptional regulation, exacerbating sodium-glucose reabsorption and glomerular hyperfiltration. This “tubuloglomerular feedback imbalance” not only elevates intraglomerular capillary pressure but also induces podocyte hypertrophy and basement membrane thickening through mTORC1 activation (82–84).

SGLT2 inhibitors (e.g., dapagliflozin) exhibit renoprotective effects beyond glucose lowering (85–87). The DAPA-CKD trial, the first renal outcome study of SGLT2 inhibitors in CKD patients (with or without type 2 diabetes), demonstrated that dapagliflozin significantly reduced: Primary composite endpoint risk (eGFR decline ≥ 50%, progression to ESRD, renal/cardiovascular death) by 39%. Renal-specific composite endpoint risk (eGFR decline ≥ 50%, progression to ESRD, renal death) by 44%. Cardiovascular composite endpoint risk (cardiovascular death or heart failure hospitalization) by 29%. All-cause mortality risk by 31% (87–90). Mechanisms include: (1) hemodynamic improvement: Inhibiting proximal tubule sodium reabsorption, activating macula densa pressure sensors, restoring tubuloglomerular feedback, and reducing intraglomerular pressure (10–15 mmHg); (2) metabolic reprogramming: Activating AMPK/SIRT1 to enhance fatty acid oxidation and reduce lipotoxicity; (3) anti-inflammatory effects: Suppressing NLRP3 inflammasome activation and lowering IL-1β/IL-18 levels (86, 87).

However, SGLT2 inhibitors may rarely induce euglycemic ketoacidosis (incidence: 0.1–0.3%), attributed to increased proximal tubule ketone reabsorption and pancreatic α-cell glucagon dysregulation (91, 92). Dual SGLT1/2 inhibitors (e.g., sotagliflozin), which inhibit intestinal SGLT1 to reduce glucose absorption, may mitigate this risk but require further validation (93–95).

#### 4.1.2 Epithelial sodium channel (ENaC)

The epithelial sodium channel (ENaC), also known as the amiloride-sensitive sodium channel (ASSC), is a Na^+^-permeable ion channel located in the apical membranes of epithelial cells in the kidney, lung, colon, and other tissues. ENaC-mediated Na^+^ transport is critical for regulating salt and water absorption across epithelial surfaces (1, 2, 8–10, 15). Composed of α, β, and γ subunits, ENaC in the distal nephron (distal convoluted tubule and collecting duct) mediates aldosterone-dependent sodium reabsorption (15–17). Hyperglycemia alters ENaC gating, disrupting renal electrolyte homeostasis, exacerbating hypertension, and accelerating DKD progression (15–17, 96). In DKD, mineralocorticoid receptor (MR) overactivation phosphorylates ENaC via serum/glucocorticoid-regulated kinase 1 (SGK1), increasing membrane expression and open probability, leading to sodium retention and elevated blood pressure (97). Additionally, ROS suppress Nedd4-2, a ubiquitin ligase responsible for ENaC γ-subunit degradation, further enhancing channel activity (15–17, 96–98).

Mineralocorticoid Receptor Antagonists (MRAs) targeting the ENaC-MR axis offer precision therapy for DKD. Traditional steroidal MRAs (e.g., spironolactone, eplerenone) showed limited long-term renal benefits and safety concerns, prompting the development of non-steroidal MRAs. Finerenone, a selective non-steroidal MRA, significantly reduces: Major endpoint incidence (ESRD, sustained eGFR decline, or death). Secondary endpoints (cardiovascular death, non-fatal myocardial infarction, non-fatal stroke, or heart failure hospitalization); with a lower incidence of hyperkalemia compared to steroidal MRAs (99–102). Renoprotective mechanisms include: (1) anti-fibrotic effects: Downregulating TGF-β1/Smad3, profibrotic growth factors (CCN2, CTGF); (2) endothelial protection: Restoring NO bioavailability and reducing endothelin-1 levels. Finerenone also alleviates macrophage overactivation via regulation of the complement C5a-C5aR1 axis (G protein subunit alpha i2) and ameliorates high glucose-induced podocyte epithelial-mesenchymal transition through Krüppel-like factor 5 (KLF5) modulation (103–105).

### 4.2 Calcium channels

The pathogenesis of DKD involves a complex interplay between calcium signaling imbalance and energy metabolism dysregulation, synergistically driving renal injury progression. Podocytes, as critical components of the glomerular filtration barrier, develop ion channel abnormalities—such as disrupted mitochondrial-endoplasmic reticulum calcium cycling and cytoskeletal dynamics—that underlie proteinuria. Hyperglycemia-induced intracellular calcium overload activates calcium-dependent proteases (e.g., calpain) and TRPC6 channels, leading to mesangial extracellular matrix deposition and podocyte cytoskeletal collapse, which exacerbate proteinuria and glomerulosclerosis (1, 2, 8–13, 19). Concurrently, endoplasmic reticulum calcium dyshomeostasis and mitochondrial calcium overload trigger oxidative stress and inflammatory responses, promoting apoptosis of renal tubular epithelial cells (1, 2, 8, 11, 12, 19).

At the metabolic level, mitochondrial dysfunction (e.g., excessive ROS production, impaired fatty acid oxidation) and AMPK/mTOR pathway imbalance induce cellular energy crises. Enhanced glycolysis exacerbates microenvironment acidosis and metabolic memory effects, further accelerating fibrosis (1, 2, 10, 13, 19). Notably, calcium signaling and energy metabolism form an intersecting regulatory network via mitochondria-associated ER membranes (MAMs) and calcium-dependent metabolic enzymes (e.g., pyruvate dehydrogenase, PDH), where oxidative stress and NF-κB-driven inflammation create a self-perpetuating cycle that amplifies renal damage. Therapeutic strategies targeting voltage-gated calcium channels (VGCCs), TRP channels, AMPK activation, mTOR inhibition, or mitochondrial antioxidants show potential clinical value. Future research should explore multi-target interventions to disrupt this pathological loop, advancing precision therapy for DKD.

#### 4.2.1 Transient receptor potential (TRP) channels

The TRP channel family (e.g., TRPC5, TRPC6, TRPV4) plays a central role in podocyte injury in DKD (18, 29, 50, 80, 106). Hyperglycemia activates angiotensin II (Ang II)/AT1R signaling, upregulating TRPC6 expression and inducing pathological calcium influx (intracellular Ca^+^ concentration > 500 nM), which drives the following pathological effects: Podocyte cytoskeletal disintegration: Calcium-dependent protease calpain cleaves synaptopodin and nephrin, disrupting slit diaphragm integrity; Mitochondrial damage: Calcium overload opens mitochondrial permeability transition pores (mPTP), reducing ATP synthesis by 50–70%. Apoptotic signaling: Calcium/calmodulin -dependent kinase II (CaMKII) phosphorylates FOXO1, suppressing anti-apoptotic genes (e.g., *Bcl-2*) (28, 106, 107). Salemkour et al. demonstrated that dual inhibition of TRPC6 and calpain restores glomerular autophagy flux, reducing renal injury with high clinical translational potential (50). Additionally, the TRPC5 inhibitor GFB-887 is under investigation for safety and efficacy in focal segmental glomerulosclerosis, refractory minimal change disease, and DKD (28).

#### 4.2.2 Voltage-gated calcium channels (VGCCs)

VGCCs mediate calcium influx during membrane depolarization and are essential for neurotransmission, muscle contraction, membrane excitability, synaptic plasticity, and gene expression (108). In DKD, hyperglycemia aberrantly activates L-type VGCCs (e.g., Cav1.2) in mesangial cells, increasing calcium current density (2–3 fold) and driving fibrosis via: (1) inflammatory cytokine release: Calcium signaling activates nuclear factor of activated T cells (NFAT), promoting TGF-β1, connective tissue growth factor (CTGF), and IL-6 synthesis; (2) extracellular matrix (ECM) deposition: Upregulating collagen IV and fibronectin expression; (3) cellular proliferation: Activating ERK1/2 pathways to induce pathological mesangial cell hyperplasia (108). Peng et al. revealed that low-dose nifedipine can enhance EPCs’ angiogenic potential and implied that chronic treatment with low-dose nifedipine may be a safe and economic manner to prevent ischemic diseases in diabetes (109). Preclinical and clinical studies suggest that L-type calcium channel blockers (e.g., manidipine, nifedipine) mitigate renal dysfunction, highlighting the need for kidney-specific VGCC modulators (110).

### 4.3 Potassium ion channels

Potassium ion channels play a pivotal role in electrolyte balance and urine concentration in the kidneys, predominantly localized in renal tubular epithelial cells (e.g., principal cells of the distal convoluted tubule and collecting duct). By regulating potassium secretion and reabsorption (e.g., ROMK channel-mediated potassium secretion), these channels maintain serum potassium homeostasis. Under pathological conditions, dysfunctional potassium channels (due to genetic mutations, pharmacological inhibition, or chronic kidney disease) may lead to hyperkalemia (impaired excretion) or hypokalemia (excessive loss), triggering systemic physiological abnormalities.

#### 4.3.1 Renal outer medullary potassium channel (ROMK)

ROMK, located on the apical membrane of the thick ascending limb (TAL) of the loop of Henle, mediates potassium recycling to sustain NKCC2 (Na^+^-K^+^-2Cl^–^ cotransporter) activity, which is critical for the urine-concentrating mechanism (1, 2, 8–10). Hyperglycemia suppresses ATP-sensitive potassium channel (KATP) opening via the AGEs-RAGE pathway, inducing depolarization of renal tubular epithelial cell membranes, mitochondrial electron transport chain uncoupling, and a 3–5-fold increase in ROS production. ROS further activates the NLRP3 inflammasome, promoting IL-1β and IL-18 release and establishing an “oxidative stress-inflammation” cascade. This cascade activates NF-κB and TGF-β1 signaling pathways, driving macrophage infiltration and collagen deposition (111). Single-cell RNA sequencing has revealed significant downregulation of fatty acid oxidation-related genes and upregulation of injury markers (e.g., Havcr1, Vcam1) and pro-inflammatory factors (e.g., SPP1) in proximal tubular cells of DKD patients, suggesting that ion channel dysfunction exacerbates renal microenvironment deterioration via metabolic reprogramming and dysregulated intercellular communication (112, 113). In DKD, hyperglycemia phosphorylates ROMK at the S44 residue (2-fold increase in phosphorylation) via the PKC pathway, leading to channel internalization and degradation. Consequences include: (1) impaired potassium secretion: Elevated serum potassium (> 5.0 mmol/L) activates ENaC, aggravating sodium retention. (2) TAL dysfunction: Reduced NKCC2 activity diminishes urinary concentrating capacity, worsening polyuria (96). ROMK gene (KCNJ1) mutations cause Bartter syndrome type II (hypokalemic alkalosis), whose pathological phenotype mirrors ROMK inhibition in DKD as a “mirror phenomenon.” The lack of selective ROMK inhibitors has hindered therapeutic exploration. Single-cell electrophysiology combined with CRISPR screening has identified aberrant crosstalk between ROMK and Kir4.1/5.1 channels in DKD renal tubular cells, suggesting the need for multi-target potassium channel modulators to restore electrolyte homeostasis (114).

### 4.4 Chloride ion channels

Chloride channels are widely distributed in glomerular and tubular epithelial cells, regulating cell volume, transmembrane potential, ion transport, and signaling (79, 114). Emerging evidence highlights the critical role of chloride channels (ClCs) in DKD pathogenesis via ion dysregulation, inflammation, oxidative stress, and fibrotic signaling. In diabetes, hyperglycemia-induced mitochondrial dysfunction and endoplasmic reticulum (ER) stress impair ClC activity (e.g., downregulated CLC-5 in proximal tubules), disrupting lysosomal acidification and protein degradation, thereby exacerbating cellular injury (115). Dysfunctional ClCs, including calcium-activated TMEM16A (ANO1) and volume-regulated VRAC (LRRC8), trigger NLRP3 inflammasome activation, excessive ROS production, and TGF-β/Smad-driven epithelial-mesenchymal transition (EMT), promoting glomerulosclerosis and interstitial fibrosis (116). Notably, TMEM16A upregulation in diabetic models correlates with elevated pro-inflammatory cytokines and extracellular matrix deposition, while its inhibition ameliorates proteinuria and renal damage (116). Impaired Cl^–^ transport via CLC-5 disrupts autophagic flux, accelerating podocyte apoptosis. These channels also modulate hemodynamic stress by influencing renal vascular tone and sodium retention (117). Additionally, the cystic fibrosis transmembrane conductance regulator (CFTR), a cAMP-activated ATP-gated Cl^–^ channel, has recently been linked to DKD pathology. Studies suggest CFTR upregulation suppresses the Wnt/β-catenin pathway, improving tubular lesions during DKD (115, 117). Although challenges remain in subtype selectivity and long-term safety, targeting specific ClCs (e.g., TMEM16A inhibitors or CLC-K antagonists) shows therapeutic promise in preclinical studies. Spatiotemporal regulation of ClCs may unveil novel strategies to mitigate DKD progression (118).

### 4.5 Aquaporins

Aquaporins (AQPs), a family of membrane proteins regulating transmembrane water and solute transport, are integral to diverse physiological processes (119, 120). Recent evidence underscores their role in DKD pathogenesis, particularly in water homeostasis, metabolic dysregulation, and cellular injury (119–121). In diabetes, hyperglycemia-induced AQP1 downregulation in proximal tubules disrupts water reabsorption, exacerbating tubular dysfunction and proteinuria. Impaired AQP7 expression in adipocytes contributes to dyslipidemia and lipotoxicity, while defective glycerol transport via AQP7 aggravates insulin resistance and ectopic lipid deposition, linking metabolic imbalance to DKD progression (122). Preclinical studies demonstrate that berberine-mediated PI3K/Akt activation restores AQP1, improving renal function in diabetic models (123). Single-cell sequencing by Tsai et al. identified AQP4 as a key mediator of early DKD signaling and intercellular crosstalk (124). Despite progress, the molecular mechanisms of AQP-mediated renal protection remain incompletely defined, necessitating further research into tissue-specific regulatory networks and translational strategies targeting AQPs to alleviate DKD.

### 4.6 Mechanosensitive ion channels

DKD is characterized by glomerulosclerosis, mesangial expansion, and proteinuria, with podocyte injury playing a central role. Podocytes endure significant mechanical stress (e.g., circumferential wall stress, filtration slit shear stress, and Bowman’s capsule fluid shear stress) due to their anatomical structure (125). Piezo proteins (Piezo1/2), key mammalian mechanotransducers, convert mechanical stimuli into intracellular chemical signals, regulating diverse physiological processes and disease progression (126). Given their mechanosensory properties and DKD pathology, Piezo1 likely acts as a critical mediator linking mechanical stress (e.g., glomerular hypertension, hemodynamic shear forces) and metabolic disturbances to downstream inflammatory, oxidative, and fibrotic pathways. Analogous to fibrosis in liver, lung, and intestine, glomerular hyperfiltration-induced mechanical stress (e.g., shear forces, intraglomerular hypertension) may activate Piezo1 in endothelial cells and podocytes, triggering sustained Ca^2+^ influx. This activates the ROS-NLRP3 inflammasome axis, promotes IL-1β release, and synergizes with TGF-β/Smad signaling to drive EMT and extracellular matrix deposition, accelerating renal fibrosis (126–130). Additionally, metabolic disturbances (e.g., lipotoxicity) may enhance Piezo sensitivity via altered membrane fluidity, amplifying oxidative-inflammatory cascades, podocyte detachment, and filtration barrier disruption. Notably, aberrant Piezo1 activation in immune cells (e.g., platelets, neutrophils) exacerbates microthrombosis and NETosis-related inflammation, forming a “metabolic-mechanical-immune” network (131, 132). Li et al. reported Piezo1 upregulation in DKD, where the Piezo1/NFATc1/TRPC6 axis promotes podocyte injury. Podocyte-specific Piezo1 knockout attenuates DKD progression (133). Current studies show that selective inhibitors (e.g., GsMTx4) or genetic targeting of Piezo1 ameliorates proteinuria, podocyte injury, and fibrosis in diabetic and hypertensive models. However, tissue-specific strategies (e.g., avoiding systemic bleeding risks) and dynamic effects across disease stages require further exploration (133, 134). Integrating mechanobiology and metabolomics may elucidate spatiotemporal regulation of Piezo1 in renal cells, offering novel targets for DKD intervention.

### 4.7 Voltage-dependent anion channels (VDAC)

VDACs (voltage-dependent anion-selective channels), also termed mitochondrial porins, are the most abundant proteins in the outer mitochondrial membrane (OMM). They mediate ion (e.g., Ca^2+^) and metabolite (e.g., ATP, tRNA, DNA) exchange between mitochondria and the cytosol, ensuring mitochondrial complex function and energy production (135). In DKD, upregulated expression of the ER-resident protein reticulon-1A (RTN1A) exacerbates tubular epithelial cell (TEC) injury via ER stress. TEC-specific RTN1A overexpression worsens DKD phenotypes, including tubular damage, interstitial fibrosis, and renal dysfunction. Mechanistically, RTN1A disrupts ER-mitochondria contacts by interacting with mitochondrial hexokinase-1 (HK-1) and VDAC1, dissociating their binding. HK-1/VDAC1 dissociation activates apoptosis and inflammasome pathways, driving TEC loss (136).

### 4.8 Toward precision ion channel therapeutics

The aforementioned ion channels play central roles in DKD pathogenesis by modulating glomerular function, podocyte homeostasis, and mitochondrial metabolism. Non-selective cation channels (e.g., TRPV4, TRPC5) drive endothelial injury and podocyte apoptosis via calcium overload, oxidative stress, and inflammation, while mitochondrial channel dysfunction exacerbates metabolic imbalance (76–81). Emerging precision strategies include: (1) Small-molecule inhibitors: Targeting channel activity (e.g., TRPV4 antagonists) or upstream regulators (e.g., finerenone for mineralocorticoid receptors) to improve proteinuria and renal function. (2) Multi-target therapies: Synergistically regulating metabolic and ionic homeostasis (e.g., SGLT2 inhibitors combined with TRPC6 antagonists). (3) Biomarker-guided approaches: Leveraging urinary exosomal miRNAs (e.g., miR-29c) or DNA methylation signatures for early diagnosis and treatment stratification.

Advanced delivery systems: Utilizing kidney-targeted nanoparticles or engineered exosomes to enhance therapeutic specificity. Despite progress, key challenges remain: (1) deciphering crosstalk between ion channel networks and metabolic/inflammatory pathways. (2) Validating clinical efficacy of novel targets (e.g., Piezo1, VDAC1) in diverse patient cohorts. (3) Optimizing personalized regimens through multi-omics integration (genomics, proteomics, metabolomics) (137–139).

By bridging mechanistic insights with scalable clinical tools, ion channel-targeted therapies are transitioning from disease delay to pathomechanistic reversal, offering a transformative framework for precision nephrology.

## 5 Current challenges and future directions

Despite significant progress in ion channel-targeted therapies for DKD, clinical translation faces multiple challenges. Current strategies have evolved from single-receptor blockade to multidimensional precision interventions. Future efforts should focus on: (1) Subtype-selective drug design: Leveraging cryo-electron microscopy (cryo-EM) and AI-based predictions to resolve dynamic channel conformations. (2) Spatiotemporal-specific modulation: Utilizing nanotechnology for localized, high-concentration drug delivery to renal tissues. (3) Multi-target synergistic interventions: Combining SGLT2 inhibitors, MRAs, and TRP antagonists to overcome compensatory signaling activation. These strategies aim to shift DKD treatment paradigms from “disease delay” to “pathological reversal.” This section systematically analyzes current limitations—spanning technical bottlenecks, safety optimization, and therapeutic innovation—and proposes a breakthrough tripartite strategy integrating precision medicine, multi-omics technologies, and optimized drug safety.

### 5.1 Integration of precision medicine and multi-omics

The convergence of precision medicine and multi-omics technologies is advancing DKD intervention toward cell-specific and dynamic regulation. Single-cell sequencing and spatial transcriptomics reveal upregulated SGLT2 and ENaC expression in proximal tubular epithelial cells and abnormal TRPC5/TRPC6 ratios in podocytes of DKD patients, highlighting the need for subtype/cell-specific inhibitors (140–143). Metabolomics identifies branched-chain amino acid (BCAA) accumulation as a driver of mitochondrial dysfunction via KATP channel inhibition (74). Machine learning algorithms are increasingly applied to integrate multi-omics data, systematically capturing complex interactions and establishing robust data linkages. AI-driven multi-omics platforms can decode “ion channel-metabolite-epigenetic” networks, enhancing patient stratification and therapeutic efficacy (144). To mitigate systemic side effects (e.g., ketoacidosis with SGLT2 inhibitors, renal limitations of non-steroidal MRAs), novel delivery systems—such as kidney-targeted nanocarriers (e.g., PEGylated liposomes achieving 10-fold higher renal drug concentration) and exosomes—enable precise delivery, minimizing off-target effects (145–147). Furthermore, multi-target therapies (e.g., SGLT2 inhibitors combined with finerenone) demonstrate synergistic benefits by dual inhibition of sodium reabsorption and TGF-β1/Smad3 signaling, reducing UACR. However, cross-scale models (e.g., PhysiCell) are required to quantify synergy and toxicity risks (148, 149). Future studies must integrate multi-omics data with dynamic pharmacodynamic models to transition DKD treatment from “single-target intervention” to “spatiotemporal precision modulation.”

### 5.2 Cutting-edge technological innovations

Cutting-edge research is driving systemic innovation from molecular mechanisms to clinical paradigms, primarily manifested in the following aspects:

#### 5.2.1 Structure-guided drug design

Cryo-electron microscopy (cryo-EM)-resolved TRPC5-calmodulin complexes, combined with AI-driven molecular dynamics simulations (e.g., AlphaFold 3), empower structural pharmacology to develop conformation-selective inhibitors. For instance, non-competitive antagonists targeting TRPC5 allosteric sites are being designed to circumvent cardiovascular side effects associated with traditional pore-blocking agents (150, 151).

#### 5.2.2 Dynamic channel network analysis

Integration of single-cell multi-omics with optogenetic techniques (e.g., ChR2 optogenetic systems) enables real-time dissection of ion channel dynamics. Light-activated modulation allows precise activation or inhibition of specific channels, achieving spatiotemporal control over channel behavior and revealing their regulatory patterns (152).

#### 5.2.3 Advanced drug screening platforms

Kidney organoid co-culture systems coupled with microelectrode arrays replicate DKD pathological microenvironments and monitor ionic current fluctuations in real time, significantly enhancing drug screening efficiency. Concurrently, machine learning models (e.g., AlphaFold 3) accelerate virtual drug discovery by predicting dynamic ion channel conformations (151, 153).

### 5.3 Toward precision spatiotemporal modulation

The interdisciplinary integration of these technologies propels DKD therapeutics from “static target inhibition” to “precision spatiotemporal modulation.” Ion channel network regulation offers a novel strategy to disrupt the “metabolic-inflammatory-fibrotic” cycle in DKD. While targeted therapies (e.g., SGLT2 inhibitors, TRP antagonists) show clinical promise, challenges persist in subtype selectivity, delivery efficiency, and patient heterogeneity. Future integration of cryo-EM structural insights, single-cell multi-omics, and AI predictive analytics will unravel channel regulatory networks, enabling patient-tailored regimens. Through global collaborations (e.g., the DKD Channelome Initiative), standardized omics pipelines, validated biomarkers, and ethical AI guidelines can democratize access to precision nephrology, ultimately transitioning DKD care from delayed intervention to pathological reversal.

## 6 Discussion and perspectives

### 6.1 Core pathogenic role of ion channel dysregulation

The pathological progression of DKD is fundamentally driven by a vicious cycle of ion channel network dysfunction and “metabolic-inflammatory-fibrotic” cascades. This review systematically demonstrates that ion channel dysregulation—via calcium overload, oxidative stress, and inflammatory microenvironments—serves as a core driver of podocyte injury, glomerulosclerosis, and interstitial fibrosis (1, 2, 9–14). Clinically validated therapies targeting SGLT2, ENaC, and TRP channels (e.g., dapagliflozin, finerenone, GSK2193874) have marked a paradigm shift in DKD management from “symptom control” to “mechanism-based intervention” (82–92, 99–102).

### 6.2 Critical bottlenecks for pathological reversal

However, achieving pathological reversal requires overcoming the following bottlenecks and advancing interdisciplinary innovation.

#### 6.2.1 Molecular mechanism elucidation

Transitioning from static targets to dynamic networks, the spatiotemporal specificity and regulatory dynamics of ion channels are central to DKD complexity. Cryo-EM and single-cell multi-omics technologies can unravel “channel-metabolism” crosstalk as novel therapeutic targets.

#### 6.2.2 Therapeutic strategy innovation

Establishment of a three-tiered precision intervention framework. (1) Early-stage intervention (high-risk screening): AI models integrating ion channel gene polymorphisms and metabolic biomarkers to predict critical targets and identify high-risk individuals. (2) Mid-stage reversal (targeting core pathology): Precision strategies for renal-targeted drug accumulation. (3) Late-stage synergy (multi-pathway blockade): Combinatorial therapies to enhance treatment efficacy.

#### 6.2.3 Technological challenges and interdisciplinary breakthroughs

(1) Drug delivery limitations: Current nanocarriers (e.g., liposomes, gold nanoparticles) exhibit ∼40% renal targeting efficiency, with unresolved long-term biocompatibility issues (e.g., hepatosplenic accumulation toxicity) (145–147). In contrast, exosomes—with superior biocompatibility, target specificity, and extended half-life—are emerging as promising delivery vehicles. Engineered exosomes modified with renal tubular-specific markers significantly enhance targeting efficiency (146, 147). (2) Dynamic monitoring: Traditional electrophysiology fails to track ion channel activity in live tissues. Novel techniques, such as optogenetics (e.g., ChR2-mediated KATP channel activation) combined with two-photon microscopy, enable real-time monitoring of podocyte calcium signaling and filtration barrier permeability (152).

### 6.3 Future directions: bridging discovery to clinical impact

Precision drug design, powered by cryo-EM structural insights and AI-driven molecular dynamics (AlphaFold 3), will yield conformation-selective inhibitors (150–153). Global consortia like the DKD Channelome Initiative aim to standardize omics pipelines, validate target engagement biomarkers, and establish ethical AI guidelines for vulnerable populations.

Future research should prioritize precision medicine approaches to bridge ion channel discoveries and clinical applications. First, artificial intelligence-driven multi-omics integration—incorporating urinary exosomal RNA profiles, single-cell epigenetic data, and wearable device metrics—will enable prediction of individual channelopathies (e.g., concurrent TRPC6/Piezo1 dysregulation) and optimization of SGLT2 inhibitor therapeutic responses. Simultaneously, biodegradable nanocarriers (e.g., megalin-targeted PLGA nanoparticles) and customized exosomes (e.g., CD133-engineered vesicles for glomerular targeting) could improve renal drug delivery efficiency while reducing off-target effects. Second, international collaborations such as the DKD Channelome Initiative should establish standardized biomarker protocols (e.g., TRPV4 autoantibody detection) for diverse populations, resolving genetic variability while ensuring target validation through blockchain-protected data exchange. Advanced structural biology tools (cryo-EM and AlphaFold 3) will elucidate glucose-altered ion channel architectures to develop highly selective (> 100-fold) allosteric drugs, complemented by patient-derived organoid-microfluidic systems for customized drug testing. Ultimately, innovative trial designs integrating computational patient avatars and adaptive Bayesian methods could halve DKD progression rates by 2,030 via coordinated modulation of metabolic, inflammatory, and fibrotic pathways.

### 6.4 Multitarget regulation by traditional chinese medicine (TCM) in DKD management

Traditional Chinese Medicine (TCM) and integrated TCM-Western medicine strategies exhibit substantial therapeutic potential in the diagnosis and management of DKD. As a central manifestation of diabetic microvascular complications, DKD necessitates combined interventions targeting metabolic regulation and renal protection. TCM modulates multiple pathological pathways in DKD: Alpiniae Oxyphyllae Fructus ameliorates inflammation and oxidative stress by activating podocyte autophagy, regulating non-coding RNAs, and restoring gut microbiota balance (154, 155). Poria cocos and its active components attenuate renal fibrosis through TGF-β1/Smad and NF-κB signaling pathway inhibition (156). Jingui Shenqi Pills, when combined with Western medications, reduce urinary albumin-to-creatinine ratio (ACR), modulate gut microbiota composition (e.g., increasing Prevotella abundance), and suppress inflammatory cytokines such as IL-2 (157). TCM also corrects epigenetic dysregulation (e.g., suppressing DNMT1-mediated DNA methylation), potentially reversing “metabolic memory” to delay DKD progression (158, 159).

In contrast, SGLT2 inhibitors (e.g., dapagliflozin) primarily inhibit proximal tubular sodium-glucose reabsorption, ameliorate glomerular hyperfiltration, and exert metabolic (e.g., glycemic and blood pressure control) and anti-inflammatory or anti-fibrotic effects. Clinical trials confirm their efficacy in slowing end-stage renal disease progression, independent of glucose-lowering mechanisms. While both TCM and SGLT2 inhibitors demonstrate renoprotective properties, TCM’s multi-dimensional regulation (e.g., epigenetics, microbiome) may address broader pathological networks, whereas SGLT2 inhibitors predominantly improve hemodynamic abnormalities.

Integrated therapies further enhance DKD management: Early-stage DKD patients benefit from combined angiotensin system inhibitors (ACEI/ARB), SGLT2 inhibitors (e.g., dapagliflozin), and TCM formulas (e.g., Tang Shen Fang), which synergistically reduce proteinuria, improve metabolic profiles, and restore gut microbiota homeostasis (160). High-risk patients with nephrotic-range proteinuria achieve superior renal outcomes (12-month proteinuria reduction: 7,289.25 mg vs. 4,512.79 mg) and fewer glycemic fluctuations with low-dose multi-target immunosuppressive regimens (glucocorticoids + tacrolimus + mycophenolate mofetil) compared to cyclophosphamide-based protocols (161).

Mechanistic studies reveal that Astragaloside IV alleviates podocyte oxidative injury via JNK/ERK1/2 pathway suppression, triptolide inhibits complement C5b-9 deposition through p38MAPK modulation, and Panax notoginseng oral liquid activates Nrf2/HO-1 to enhance antioxidant defenses, collectively preserving renal function (159). These advancements underscore the promise of targeting “metabolic memory” and multi-pathway interventions for DKD, warranting further validation of TCM’s mechanistic precision and long-term safety in multicenter trials.

## 7 Conclusion

**FIGURE 2:**
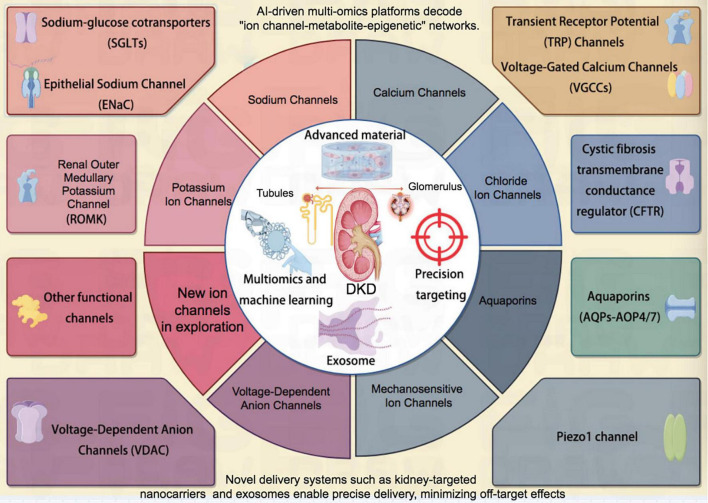
Schematic diagram of a series of ion channels related to DKD. DKD, diabetic kidney disease.

In conclusion, ion channel network modulation offers a novel therapeutic paradigm for disrupting the “metabolic-inflammatory-fibrotic” vicious cycle in DKD. While targeted therapies (e.g., SGLT2 inhibitors and TRP channel antagonists) demonstrate clinical efficacy, persistent challenges remain regarding subtype selectivity, drug delivery efficiency, and patient heterogeneity. The convergence of cryo-electron microscopy (cryo-EM), single-cell multi-omics profiling, and AI-based predictive modeling will elucidate comprehensive channel regulatory networks, while parallel exploration of traditional medicine compounds may enable truly personalized therapeutics (Figure 2). Through multidisciplinary collaboration and innovative pharmacologic development, we can potentially transform DKD management—from merely delaying disease progression to achieving pathological reversal of ion imbalance-induced renal injury.
